# Multi-tissue transcriptomic profiling of foxtail millet in response to drought and rewatering

**DOI:** 10.1186/s12870-026-08906-y

**Published:** 2026-05-11

**Authors:** Kai Zhang, Wenbing Wang, Shuke Deng, Junrong Li, Qi Wang, Bing Xiang, Yaying Yan, Pei Wu, Guohan Wu, Zhen Liang

**Affiliations:** 1 School of Life Science, Key Laboratory of Sustainable Dryland Agriculture of Shanxi Province, Shanxi University, Taiyuan, Shanxi 030006 China; 2 Shanxi Baijiu Ecological Brewing Technology Innovation Center, Shanxi University, Taiyuan, Shanxi 030006 China

**Keywords:** Foxtail millet, Drought, Rewatering, Transcriptomics, Multi-tissue

## Abstract

**Background:**

Drought is a critical abiotic stress limiting crop productivity. However, the molecular mechanisms underlying coordinated multi-tissue responses to drought and subsequent recovery remain unclear.

**Results:**

In this study, leaf‑based physiological measurements, as well as transcriptomic sequencing, and molecular network analysis of four tissues (i.e., roots, stems, leaves, and grains) under prolonged drought from booting to maturity and short-term rewatering were performed to systematically dissect the dynamic regulatory programs during drought adaptation and post‑rewatering recovery in foxtail millet. The results showed that the H_2_O_2_, proline, and malondialdehyde contents were significantly increased under prolonged drought and decreased to near control levels after rewatering. A total of 34 584 expressed genes was detected across all four tissues, among which 4 221 and 5 844 differentially expressed genes (DEGs) correlated with drought and rewatering treatments, respectively. Gene ontology and MapMan analysis across all four tissues revealed that DEGs associated with plant hormone and photosynthesis underwent pronounced dynamic reprogramming under drought and rewatering conditions. Furthermore, we identified a conserved gene module, *HPP* - *Golden2-like* (*Seita.9G137500*- *Seita.4G270600*), that responds to both drought and rewatering. This module, identified for the first time in foxtail millet, likely coordinates drought stress signaling with rewatering‑induced repair processes.

**Conclusion:**

The study reveals a multi-tissue coordination framework for drought response and post‑rewatering recovery in foxtail millet, providing new theoretical insights and genetic resources for synergistic improvement of drought tolerance and post-drought resilience in crops.

**Supplementary Information:**

The online version contains supplementary material available at 10.1186/s12870-026-08906-y.

## Introduction

Drought, as one of the most costly and deadly environmental phenomena worldwide, has drawn extensive attention due to its severe impacts on ecosystems, economies, and human livelihoods [1]. Agricultural irrigation consumes over 70% of the Earth’s available freshwater annually, and the level of agricultural production sustained by such usage currently meets the growing demand for staple crops from an increasing global population. However, under future water-limited conditions, water demand will pose a significant challenge to the sustainable development of human societies [2, 3]. Throughout evolutionary history, plants have developed a complex set of regulatory mechanisms spanning morphological, developmental, physiological, and transcriptional strategies to respond to and mitigate damage from abiotic stresses [4, 5]. Therefore, elucidating the molecular basis of stress tolerance in crops is essential for breeding varieties that can maintain high and stable yields under unfavorable conditions.

Foxtail millet (*Setaria italica* L.), belonging to the *Panicoideae* subfamily and *Setaria* genus within the *Poaceae* family, is a grain crop highly adapted to dryland farming [6]. Beyond its nutritional richness - providing carbohydrates, proteins, and dietary fiber - it has emerged as an ideal model plant for studying plant drought-tolerance mechanisms and C4 photosynthesis due to its small genome, short life cycle, and the established genetic transformation system [7, 8]. The core advantage of foxtail millet lies in its exceptional drought tolerance, arising from synergistic multi-level traits. As a C4 plant, its photosynthetic system efficiently fixes CO₂ under high-temperature and drought conditions while minimizing stomatal transpiration, achieving high water-use efficiency [9]. Notably, its grains require only ~ 26% water for germination - far less than wheat or maize - ensuring successful seedling establishment even in arid soils [10]. Morphologically, a well-developed root system enables deep soil water extraction, while small leaf area reduces water loss. Its leaves exhibit the typical C4 Kranz anatomy, which underpins efficient photosynthesis and water conservation [11, 12]. Thick cell walls and orderly epidermal cells further enhance water retention and mechanical support [10]. Together, these integrated traits establish foxtail millet not only an important crop for food security in arid and semi-arid regions but also a valuable platform for dissecting the molecular mechanisms of plant drought tolerance and thereby improving other major cereal crops.

The impact of drought on the vegetative and reproductive growth of plants has long been a key research focus, and enhancing drought tolerance in reproductive tissues is a primary goal for breeders and the global agricultural biotechnology industry [13, 14]. The booting stage is the most critical reproductive growth phase for grain yield formation in foxtail millet and is also highly sensitive to water stress [15–17]. Drought during this stage not only directly affects the physiological functions of vegetative organs but also severely disrupts reproductive development, ultimately leading to significant reductions in grain yield and quality [18]. Therefore, elucidating how different tissues of foxtail millet coordinate their responses to drought stress during the booting stage is of central importance for a deeper understanding of its drought resistance mechanisms, guiding water-saving cultivation practices, and developing drought-tolerant varieties.

Under drought stress, the different tissues of foxtail millet do not respond in isolation but form an integrated resistance network through the transport of water, osmolytes, and signaling molecules such as abscisic acid (ABA) [15, 19, 20]. The roots, as the primary organs sensing soil water content, enhance water uptake and retention by accelerating downward growth, increasing the root-to-shoot ratio, regulating aquaporin activity, and synthesizing osmoregulatory substances such as proline, while also transmitting stress signals to the aboveground parts [21, 22]. The stems serve not only as conduits for water and solutes (including mineral nutrients and assimilates) transport but are also critical for maintaining mechanical strength to prevent lodging [23, 24]. The leaves, as the primary sites of photosynthesis and transpiration, exhibit the most direct and complex responses: stomata close rapidly to reduce water loss, while the photosynthetic apparatus mitigates photoinhibition through thermal dissipation, activation of antioxidant systems, and the efficient operation of the C4 photosynthetic pathway [25]. The developing grains, as the ultimate “sink” organs, are most profoundly affected by stress. Drought disrupts the synthesis, transport, and unloading of assimilates into the grains, leading to impaired grain filling and reduced kernel weight [26, 27]. Meanwhile, hormonal balance within the grains and the metabolic pathways for storage substances undergo reprogramming, directly affecting the final yield and nutritional quality [28].

Previous studies predominantly focused on single tissues (especially leaves) or two to three tissues (roots, stems, and leaves), examining the physiological or molecular responses to short-term drought during seedling or grain-filling stages [15, 19, 21]. However, a systematic understanding of the coordinated multi-tissue response mechanisms under prolonged drought during the critical booting stage remains limited. In the present study, we integrated multidimensional data, including physiological phenotypes and transcriptomics, from roots, stems, leaves, and grains, and analyzed their interconnected regulatory networks to provide a more comprehensive understanding of the overall principles underlying drought resistance in foxtail millet. This will offer critical theoretical foundations for enhancing drought resilience and stable yield during the reproductive stage through molecular breeding or integrated cultivation management strategies.

## Materials and methods

### Plant material, growth conditions, and treatments

Seeds of the foxtail millet (*S. italica* L.) cultivar Jingu 21, widely planted in the Shanxi province of China, were obtained from Shanxi Agricultural University, China. The experiment was carried out from June 20 to October 16, 2025 at Shanxi University, China. The seeds of Jingu 21 were germinated in pots (8 × 10 × 11 cm) with sand/vermiculite mixture (1:1, v/v) in a greenhouse (16-hour light/8-hour dark, 50 000 lx). Two-week-old seedlings were then transplanted into polyvinyl chloride pots (19 × 24 × 24 cm) filled with the same substrate mixture, with two seedlings per pot. These pots were placed on the exterior corridor of the second floor of Chongli Building at the Dongshan Campus of Shanxi University as described previously [29]. The soil moisture analyzer (Jiangren Tang, Guangzhou, China) was buried into at least three points of each pot to monitor changes in relative soil humidity dynamically. Thus, this study employed a pot-based cultivation system combined with controlled water withholding to simulate drought stress.

Until the booting stage, all pots were watered regularly to maintain approximately 70% relative soil humidity during the vegetative growth phase. About two months after sowing (August 27, 2025), most foxtail millet plants entered the booting stage. At this point, the plants were randomly divided into three groups: well-watered control group (CK), drought-stressed group (DS), and rewatered group (RW). Each group has at least nine pots. Water was then withheld from both the DS and RW groups. By September 15, 2025, all foxtail millet plants had fully booted. The relative soil humidity content in the DS and RW groups decreased to 20 ~ 30% by September 26, 2025.One month later (i.e., October 15, 2025), the RW group was subjected to rewatering treatment for one day. That is, the drought treatment was defined as the period from the cessation of irrigation (August 27th) to the day of rewatering (October 15th)-approximately 50 days, encompassing both the soil drying phase and the sustained low‑moisture phase. Then, the roots, stems, leaves, and grains from CK, DS, and RW groups were synchronously and individually collected on the next day (i.e., October 16, 2025). All samples were immediately frozen in liquid nitrogen and stored at -80 °C for subsequent analysis. Three biological replicates were included for each treatment condition.

### Measurement of physiological indicators

To estimate the physiological indicators under CK, DS, and RW conditions, fresh flag leaves from each treatment above were selected to measure the changes in foxtail millet. The hydrogen peroxide content (H_2_O_2_), proline content, and malondialdehyde (MDA) content were determined by using a commercial H_2_O_2_ content assay kit (BC3590), proline content assay kit (BC0290), and MDA content assay kit (BC0020) (Solarbio, China), respectively, according to the manufacturer’s instructions. Three biological replicates and three technical replicates were performed.

### RNA extraction, library construction, sequencing, and read mapping

For each tissue type and treatment condition, total RNA extraction, library construction, Illumina sequencing, and read mapping were performed as described in a previous study with minor modifications [30]. Briefly, total RNA was extracted from the roots, stems, leaves, and grains (with three biological replicates for each group) using the TRIzol^®^ Reagent according to the manufacturer’s protocol (Invitrogen, USA). Next, the RNA-seq libraries were constructed by following the instructions of the Illumina^®^ Stranded mRNA Prep, Ligation (Illumina, USA), and then sequenced on NovaSeq X Plus platform (PE150) (Illumina, USA) using NovaSeq Reagent Kit (Illumina, USA). The generated raw reads were trimmed and quality-controlled using by fastp (https://github.com/OpenGene/fastp, Version 0.23.4) and HISAT2 (https://daehwankimlab.github.io/hisat2/, Version 2.2.1) with default parameters. The clean reads were then separately mapped to the *S. italica* reference genome (https://phytozome-next.jgi.doe.g.ov/info/Sitalica_v2_2) by StringTie (https://ccb.jhu.edu/software/stringtie/, Version 2.2.1).

### Differential expression analysis and functional enrichment analysis

The gene expression level was quantified by TPM (transcripts per million reads), and the genes with TPM ≥ 1 were identified as expressed. Differentially expressed genes (DEGs) were identified using DESeq2 with fold change ≥ 2 and P-value < 0.05 [31]., Gene ontology (GO) functional annotation was conducted using the GO database (http://www.geneontology.org/). In addition, GO enrichment analysis was performed using Cytoscape (version 3.8.0) with the help of BiNGO and EnrichmentMap apps as described previously [30], and was presented in Adobe Illustrator (2020). Furthermore, Mapman (version 3.5.1R2) was performed for pathway enrichment analysis of different DEG sets by mapping to *Setaria italica* v2.2.protein.fa.gz.

### Gene regulatory network analysis

The co-expression network was constructed using weighted gene co-expression network analysis (WGCNA) package with the default parameters except that mergeCutHeight = 0.15, and minModuleSize = 20. The express correlation analysis was constructed to identify the gene pairs with |Spearman correlation coefficient|≥0.8 at Benjamini Hochberg-corrected P-value < 0.05. Additionally, transcriptional factors (TFs) were predicted via the Plant Transcription Factor Database (https://planttfdb.gao-lab.org/).

### Quantitative real-time PCR (qRT-PCR) validation

Total RNAs from each sample (roots, stems, leaves, and grains) were isolated and reverse transcribed to the first-strand cDNA with *EasyScript*^®^ One-Step gDNA Removal and cDNA Synthesis SuperMix (TransGen, China). To determine gene expression levels, qRT-PCR was conducted with *PerfectStart*^®^ Green qPCR SuperMix (TransGen, China) on CFX Connect Real-Time System (Bio-Rad, USA) according to the provided protocol. All the primers are list in Table S1, EF1α was selected as the reference gene in grains, leaves, and stems, while SDH as the reference gene in roots [19]. Relative gene expression levels were presented using the comparative 2^–ΔΔCT^ method. For each sample in the qRT-PCR, three biological and three technical replicates were performed.

### Relative luciferase (LUC) activity

The coding sequences of *Seita.4G270600* (*HPP*) were amplified and cloned into the pCambia1301 vector to obtain the effector constructs Ubi: Seita.4G270600. The 2 kb promoter of *Seita.9G137500* (*Golden2-like*, *GLK*) was amplified and cloned into pGreen II 0800 to generate the reporter constructs Seita.9G137500P: LUC. Relative LUC activity assays were performed in foxtail millet protoplasts as described previously [32]. Briefly, 3-week-old foxtail millet seedlings were used to extract foxtail millet protoplasts. Different combinations of constructs were co-transfected into foxtail millet protoplasts and incubated in darkness for 14 h at 25℃. Firefly LUC and renilla luciferase (REN) activities were examined with the Dual-Luciferase Reporter Assay Kit (Promega, USA) according to the instructions. Transcriptional activity was indicated by the ratio of LUC/REN, and 35 S: GFP was used as an internal control. Six biological replicates were performed for this experiment.

## Results

### The physiological responses of Jingu 21 to drought stress and subsequent rewatering

**Fig. 1:**
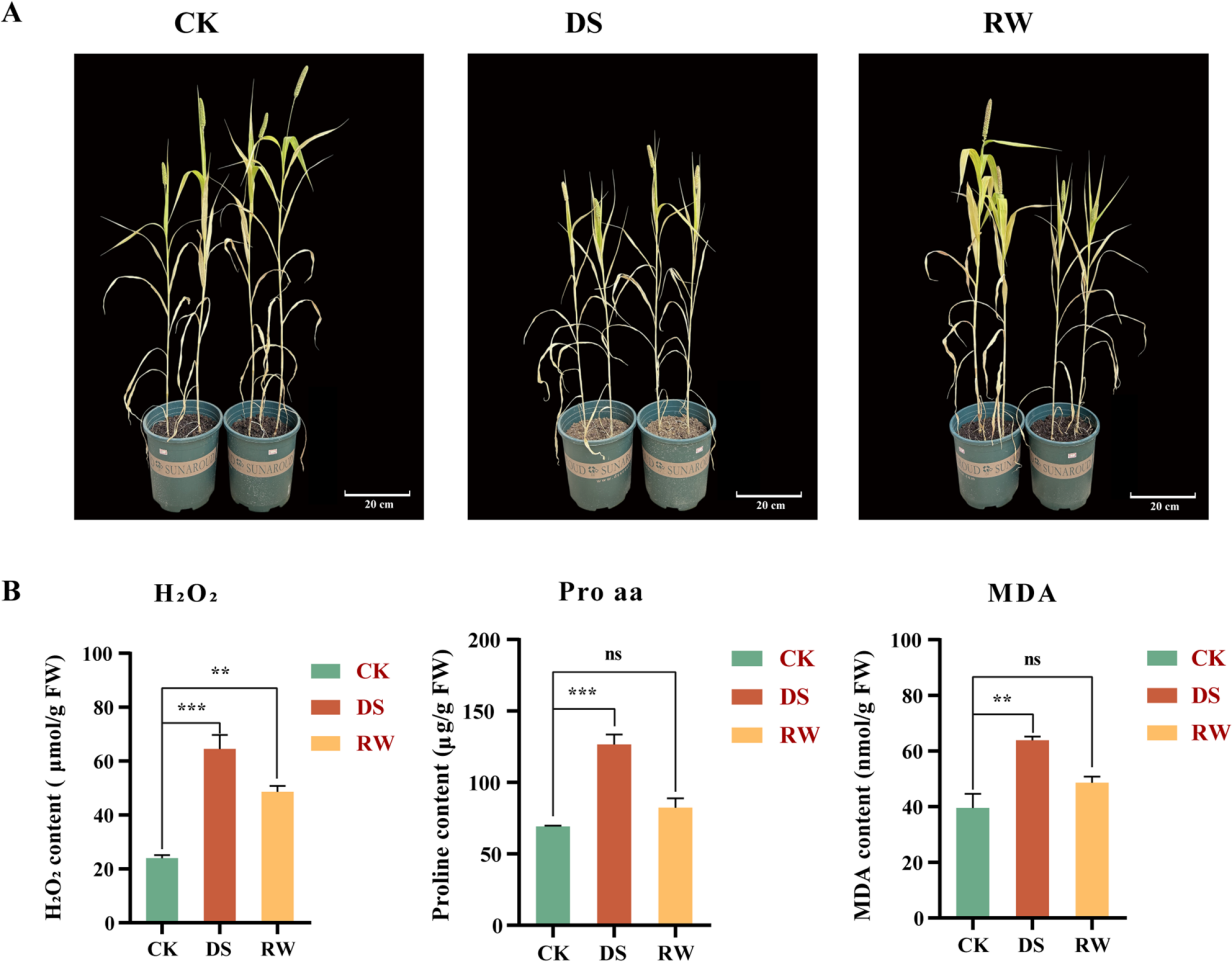
Phenotypic and physiological indexes analysis of foxtail millet under DS and RW treatments.** A** Phenotype of foxtail millet under CK, DS, and RW treatments (CK, control; DS, drought stress; RW, rewatering). Each group has at nine to ten pots. **B** Effect of drought stress and rewatering on H_2_O_2_, Proline, and MDA contents in foxtail millet leaves. All the results are represented as the mean ± standard deviation of three replicates. *p*-values were determined using the One-Way ANOVA: *p < 0.05, **p < 0.01, ***p < 0.001

Jingu 21, a foxtail millet cultivar widely cultivated in the Shanxi Province, is characterized by high yield, superior quality, and strong drought resistance. Yet it remains highly susceptible to water deficit from the booting stage to the maturity stage, which severely impacts its grain yield. To comprehensively investigate the effects of drought tolerance on foxtail millet, we mimic the drought stress during the whole booting stage to the maturity stage. As shown in Figure S1A, the relative soil humidity of CK was maintained at approximately 70% throughout the plant development, while it decreased to 20 ~ 30% in both the DS and RW groups. Following the booting of all foxtail millet plants (September 15, 2025), RW groups were rewatered one month later (October 15, 2025) for recovery. The growth of foxtail millet in the well-watered CK group was obviously better than that in the DS and RW groups (Fig. 1A). Notably, both DS and RW plants exhibited enhanced root elongation relative to the CK group (Figure S1B).

Distinct physiological responses in leaves were observed between CK, DS and RW treatments during reproductive growth stage of foxtail millet. As shown in Figs. 1B, H₂O₂ content rose from 23.93 µg/g in CK to 64.56 µg/g (a 2.70-fold increase) in DS, and declined to 48.61 µg/g, which remained 2.03-fold higher than CK. It indicates that drought-induced H₂O₂ accumulation was partially alleviated upon rehydration. Meanwhile, proline content increased from 69.19 µg/g in CK to 126.62 µg/g (a 1.83-fold increase) in DS and decreased to 82.35 µg/g in RW (a 1.19-fold increase to CK), thereby functioning as an osmotic regulator to maintain cellular turgor pressure under drought stress condition. Furthermore, MDA content in DS significantly increased from 39.57 nmol/g (CK) to 63.87 nmol/g (a 1.61-fold increase), then decreased to 48.60 nmol/g after rewatering, returning to a level close to that of the CK group. These physiological changes prompted us to investigate the underlying molecular mechanisms through transcriptome analysis.

### Global comparison of transcriptomes in different tissues under drought and rewatering conditions

**Fig. 2:**
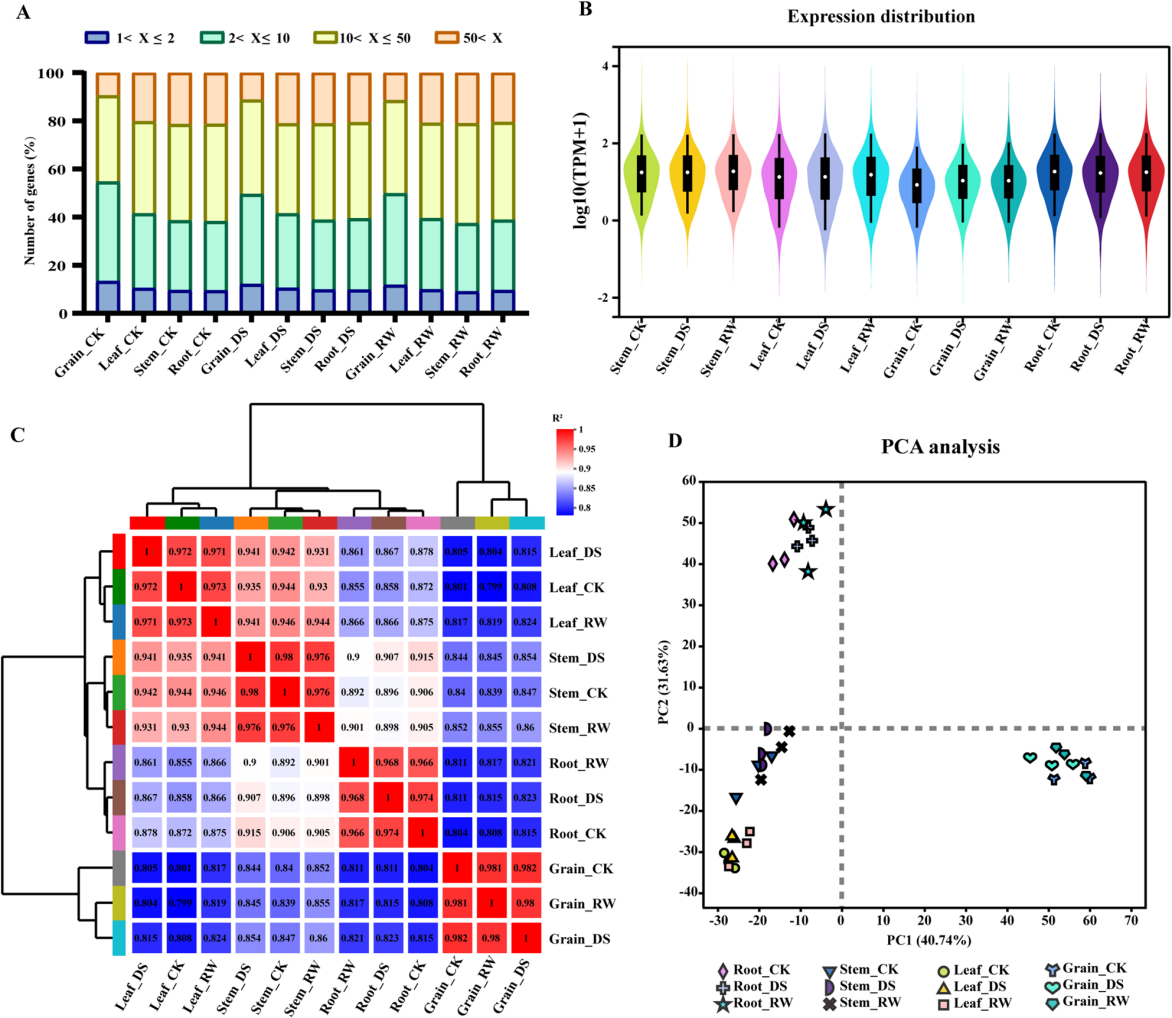
Global transcriptomic profiles of different tissues under different treatments in foxtail millet.** A** Fraction of genes expressed at different expression levels (based on TPM) in different tissues/treatments of foxtail millet in response to drought stress and rewatering. **B** Density distribution of gene expression levels. Expression levels for all genes across individual samples from root, stem, leaf, grain under CK, DS and RW treatments. **C** Heatmap analysis between transcriptomes of each tissue and each treatment from foxtail millet. SCC analysis between transcriptomes of root, stem, leaf, and grain at CK, DS, and RW treatments. **D** PCA showing clustering of transcriptomes of different foxtail millet tissues under different treatments

To elucidate the transcriptional response patterns across different tissues of foxtail millet under drought and rewatering conditions, we first conducted a global analysis of transcriptomic data from four tissues (i.e., roots, stems, leaves, and seeds) under CK, DS, and RW treatments. A total of 36 pair-end libraries were constructed for transcriptomic sequencing and the clean data of each sample reached 6.3 GB, and the total mapped bases ranged from 88.63% to 96.65% (Table S2). A total of 34 584 expressed genes was detected in all samples (Table S3). Of these, the number detected in individual tissues ranged from 17 470 (50.51% of the total) in leaf_DS to 20 012 (57.86%) in grain_RW (Figure S2). Approximately 8.92–13.27% of genes exhibited low expression levels (1 ≤ TPM ≤ 2) across the samples analyzed, a proportion comparable to that of very highly expressed genes (FPKM ≥ 50), which accounted for 9.6-21.55% (Fig. 2A). The proportion of genes with moderate expression levels (2 < FPKM ≤ 10) ranged from 28.42% to 41.35%, which was comparable to that of genes with high expression levels (10 < FPKM ≤ 50) at 35.78–41.50% (Fig. 2A). Among these, 61, 1, 2, and 191 genes exhibited stable expression under both drought or rewatering conditions and were specifically expressed in roots, stems, leaves and grains, respectively (Table S4).

The gene expression abundance distributions varied noticeably among tissues and treatments. Specifically, most genes were enriched within the log₁₀(TPM) range of 0–2 (Fig. 2B, Figure S3), which is consistent with the typical distribution pattern of transcriptomic data. Under RW, expression density curves across tissues were more concentrated in the low-expression interval yet broader in the mid‑ to high‑expression intervals, suggesting that rewatering may induce widespread activation or suppression of a subset of genes (Fig. 2B). Hierarchical clustering based on gene expression profiles showed that samples clustered primarily by tissue type (roots, stems, leaves, and grains), with further separation by treatment (CK, DS, and RW) within each tissue (Fig. 2C). Consistently, principal component analysis (PCA) further revealed that stems and leaves transcriptome of the three treatments clustered together, and showed substantial differences with roots and grains transcriptome, indicating somewhat distinct transcriptional programs between these tissues (Fig. 2D). Collectively, these results show that both prolonged drought and short-term rewatering induce systematic transcriptional changes across multiple tissues, with rewatering activating a distinct gene expression program rather than simply reversing drought‑induced responses in foxtail millet.

### Transcriptional responses to drought stress and subsequent rewatering

**Fig. 3:**
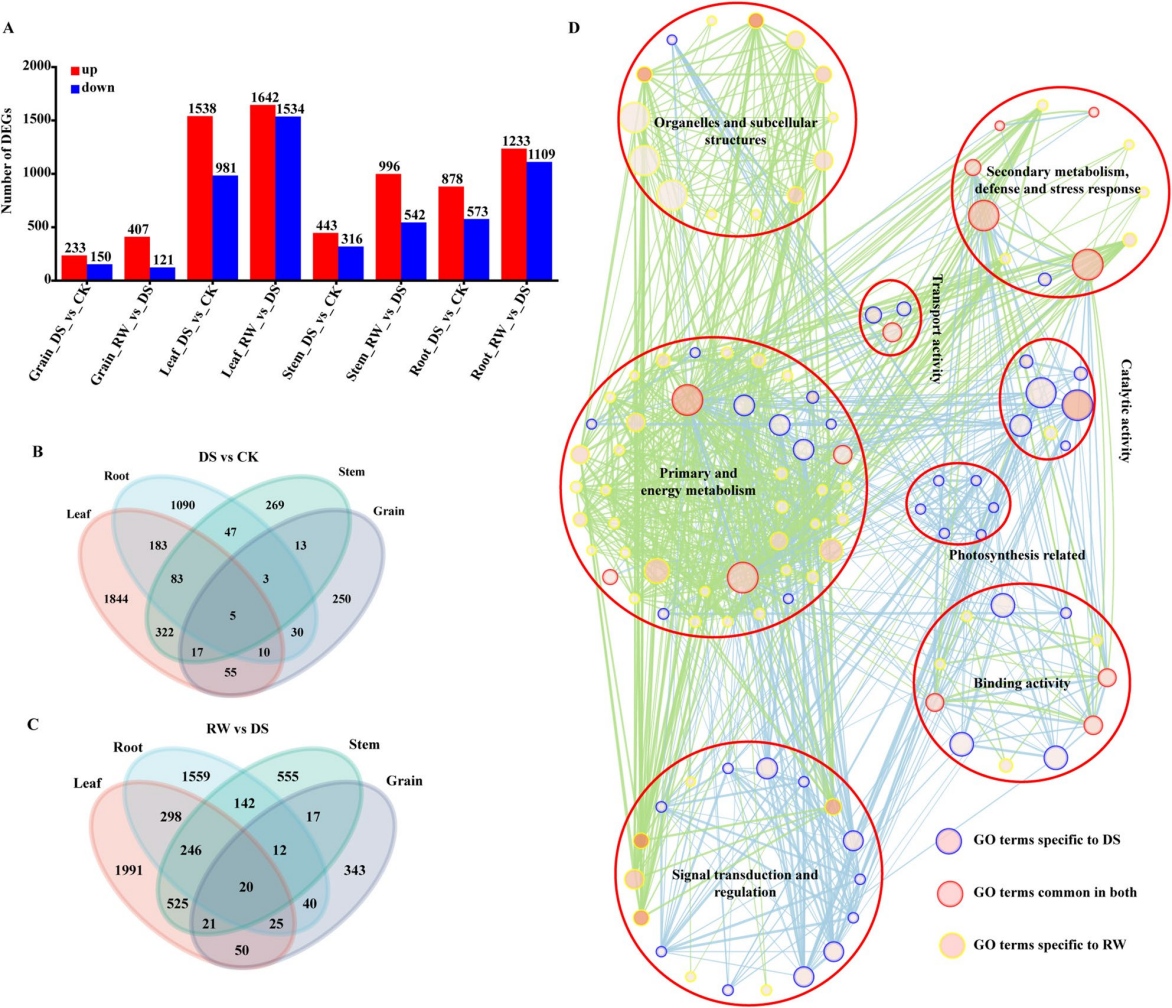
Differential gene expression in different tissues under different treatments of foxtail millet.** A** Number of up-regulated and down-regulated DEGs in root, stem, leaf, and grain in DS vs. CK and RW vs. DS, respectively. **B** Venn diagrams of the DEGs among four different tissues in DS vs. CK group. **C** Venn diagrams of the DEGs among four different tissues under RW vs. DS group. **D** GO enrichment map of preferentially DEGs from all the four tissues in DS and/or RW treatment. Significantly enriched GO terms were overlapped to highlight the terms specifically or commonly in DS and/or RW. Selected broad GO terms significantly enriched specifically or commonly in DS and/or RW have been highlighted in colored circles (blue, DS; yellow, RW; red, common). An enlarged version with GO terms labeled is provided in Figure S4

To systematically elucidate the transcriptional regulatory differences among various tissues of foxtail millet during DS and RW, DEGs were identified for the comparisons DS vs. CK and RW vs. DS, respectively. Under drought conditions (DS vs. CK), a large number of DEGs were detected in all tissues (Fig. 3A, Table S5). Among them, the number of up-regulated DEGs in leaves was the most prominent (1 538), exceeding that of down-regulated DEGs (981), whereas the numbers of up- and down-regulated DEGs in roots, stems, and grains were relatively balanced, although up-regulated DEGs still dominated (878, 443, and 233, respectively), indicating that the leaf transcriptome underwent the most extensive transcriptional reprogramming in response to drought. After rewatering treatment (RW vs. DS), the total number of DEGs increased in all tissues compared to DS vs. CK (Fig. 3A, Table S5). Notably, up-regulated DEGs remained predominant across all tissues, with the highest numbers in leaves (1 642) and roots (1 233), followed by stems (996) and grains (407), suggesting that rewatering process may involve more extensive transcriptional reprogramming in these tissues. Among these, only 5 and 20 DEGs out of 4221 and 5844 DEGs were shared in the four tissues under drought conditions and subsequent rewatering, respectively (Fig. 3B and C).

Then, an overlapping analysis of GO terms significantly enriched in each condition was conducted by generating enrichment maps, and it was found that several terms were specifically enriched either in one condition or commonly enriched in both conditions (Fig. 3D and **Figure S4**). For example, biological process GO terms related to photosynthesis and catalytic activity were specific to the drought stress condition, whereas GO terms related to organelles and subcellular structures were specific to the rewatering condition (Fig. 3D). These results indicate that drought primarily impacts photosynthetic functions, whereas rewatering engages pathways involved in subcellular remodeling. GO terms related to primary and energy metabolism and secondary metabolic processes were found enriched in both stress conditions. It may present that continuous metabolic remodeling (such as primary, secondary and energy metabolism) constitutes the core of the common adaptive strategy for plants in response to drought stress and recovery processes.

### Hormone and photosynthesis pathways are critical for drought and rewatering responses

To gain a deeper insight into the coordinated roles of the four aforementioned tissues during drought response and rewatering processes, we conducted a comparative analysis of the biological functions and metabolic pathways associated with DEGs identified in each tissue under both drought and rewatering conditions.

**Fig. 4:**
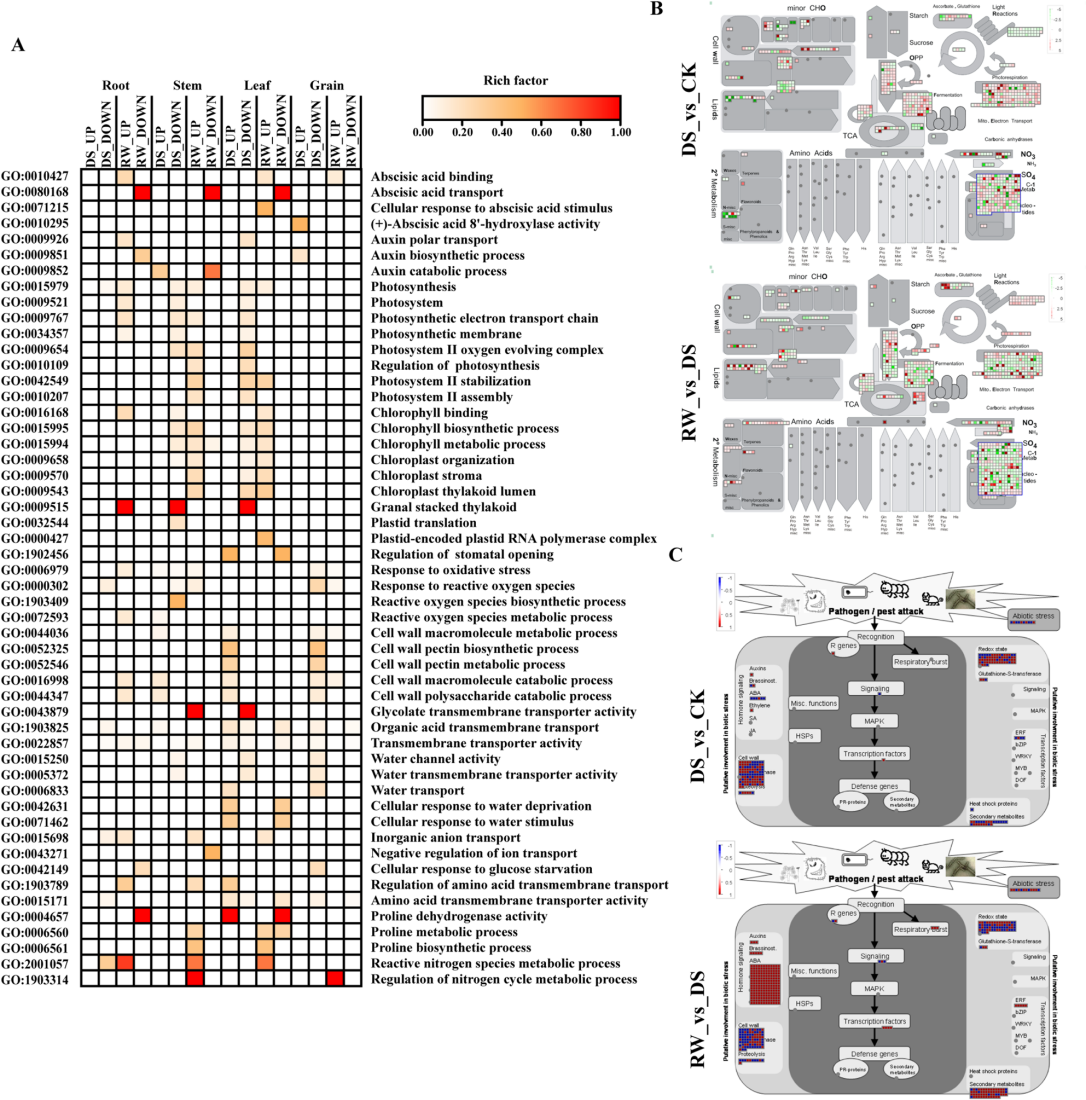
Differentially enriched pathways for the DEGs in different tissues in response to drought stress and rewatering.** A** GO pathway analysis of up- and down-regulated DEGs in root, stem, leaf, and grain in response to drought stress and subsequent rewatering, respectively. The DS_UP and DS_DOWN columns indicate enrichment results for up- and down- regulated DEGs in each tissue under DS vs. CK, respectively; and the RW_UP and RW_DOWN columns show the corresponding results for RW vs. DS. The color scale at the top represents rich factor, where higher values correspond to deeper colors and indicate greater enrichment. **B** Metabolic pathways with differential expression profiles in foxtail millet leaves under DS and subsequent RW. Red squares represent upregulated DEGs in DS vs. CK and RW vs. DS groups on the log2 scale, and green squares represent downregulation DEGs in DS vs. CK and RW vs. DS groups. **C** Biotic stress pathway analysis with differential expression profiles in foxtail millet leaves under DS and subsequent RW. Red squares represent upregulated DEGs in DS vs. CK and RW vs. DS groups on the log2 scale, and blue squares represent downregulated DEGs in DS vs. CK and RW vs. DS groups

In DS vs. CK, DEGs in the stems were significantly enriched in processes related to cell wall modification and auxin metabolism. Specifically, DEGs associated with cell wall macromolecule catabolism (GO: 0016998) and cell wall polysaccharide catabolic processes (GO: 0044347) were upregulated (Fig. 4A). Consistently, Mapman pathway analysis revealed a substantial number of DEGs distributed across cell wall related pathways, further corroborating the active remodeling of cell wall architecture under drought stress (Figure S5). And DEGs involved in auxin catabolic processes (GO: 0009852) showed increased expression in stems, potentially inhibiting elongation growth and redirecting resources toward structural reinforcement and defense (Fig. 4A). Mapman pathway analysis revealed distinct tissue‑specific patterns in photosynthetic regulation under DS condition. DEGs involved in light reactions and photorespiration were downregulated in leaves, stems, and roots, but showed enhanced expression in grains (Fig. 4B, Figure S6). Additionally, numerous DEGs associated with tetrapyrrole biosynthesis—a key pathway for chlorophyll synthesis—were substantially upregulated in leaves, stems, and roots under DS (Fig. 4B, Figure S6), likely representing a compensatory or signaling‑related response that operates concurrently with the global downregulation of photosynthetic apparatus. Consistently, GO enrichment analysis further revealed that DEGs associated with multiple photosynthesis-associated terms were significantly downregulated in leaves, including photosynthesis (GO: 0015979), photosystems (GO: 0009521), photosynthetic electron transport chain (GO: 0009767), photosynthetic membrane (GO: 0034357), and photosystem II oxygen-evolving complex (GO:0009654) (Fig. 4A). Similarly, DEGs involved in chlorophyll metabolic (GO: 0015994) and biosynthetic (GO: 0015995) processes, as well as chloroplast organization (GO: 0009658) and stromal components (GO: 0009570, GO: 0009543), were also downregulated in leaves (Fig. 4A). Concurrently, DEGs related to cell wall pectin biosynthetic (GO: 0052325) and metabolic (GO: 0052546) processes were upregulated in leaves, which may help reduce water loss through cell wall modification. Notably, DEGs involved in positive regulation of stomatal opening (GO: 1902456) were downregulated in leaves under drought, consistent with the need to minimize transpirational water loss (Fig. 4A). In grains, a unique regulatory pattern was observed for ABA metabolism. DEGs encoding (+)-abscisic acid 8’-hydroxylase activity (GO: 0010295), a key enzyme catalyzing ABA catabolism, were specifically upregulated in DS (Fig. 4A). In roots, while fewer pathways showed significant enrichment under drought compared to other tissues, DEGs related to response to oxidative stress (GO: 0006979) and ROS (GO: 0000302) were moderately modulated, indicating active management of oxidative pressure (Fig. 4A). Together, these findings demonstrate that different tissues adopt distinct yet coordinated adaptive strategies in response to drought stress.

Following short-term rewatering, the transcriptional landscape underwent a dynamic reconfiguration. Notably, Mapman analysis revealed that numerous DEGs associated with the ABA pathway were upregulated across all four tissues after rewatering (Fig. 4C, Figure S5). Consistently, GO enrichment analysis showed that DEGs involved in ABA binding (GO: 0010427) were upregulated in roots, stems, and leaves, and DEGs involved in cellular response to ABA stimulus (GO: 0071215) were specifically enhanced in leaves. In contrast, DEGs involved in ABA transport (GO: 0080168) were significantly downregulated in roots, stems, and leaves upon rewatering (Fig. 4A). This indicates a functional transition of ABA from a systemic alarm signal during drought to a local coordinator of repair processes during early recovery. Additionally, several repair-related processes of photosynthetic pathways in leaves were activated. For example, DEGs involved in photosystem II stabilization (GO: 0042549) were upregulated, alongside components of the plastid-encoded plastid RNA polymerase complex (GO: 0000427) and continued activity in chlorophyll metabolic processes (GO: 0015994, GO: 0015995). Consistently, Mapman analysis revealed that DEGs associated with light reactions and photorespiration were predominantly upregulated not only in leaves but also in roots and stems upon rewatering (Fig. 4B, Figure S6), indicating a broad, multi‑tissue activation of photosynthetic recovery. In contrast, most DEGs involved in tetrapyrrole biosynthesis were downregulated across these tissues (Fig. 4B, Figure S6), suggesting a temporary suppression of de novo chlorophyll production while repair mechanisms are prioritized. These changes suggest that the plant prioritizes repairing the most vulnerable components of the photosynthetic apparatus, such as photosystem II, before restarting the entire photosynthetic engine. Interestingly, regulation of stomatal opening (GO: 1902456) remained downregulated in leaves after rewatering, suggesting a cautious, safety-first approach that prevents premature transpiration before hydraulic and photosynthetic systems are fully reinstated. In roots and stems, DEGs involved in auxin polar transport (GO: 0009926) were upregulated in roots (Fig. 4A), indicating a rapid reconfiguration of auxin distribution that may promote the shift from deep root growth to lateral root development, optimizing water uptake from rehydrated soil. Similarly, DEGs involved in proline metabolic (GO: 0006560) and biosynthetic (GO: 0006561) processes were upregulated in stems and leaves after rewatering, suggesting active turnover of this osmoprotectant to support metabolic recovery (Fig. 4A).

Overall, these results demonstrate that prolonged drought and subsequent rewatering elicit highly tissue-specific and dynamically reprogrammed transcriptional responses in foxtail millet.

### DEGs in ABA and IAA biosynthesis and signaling pathways

**Fig. 5:**
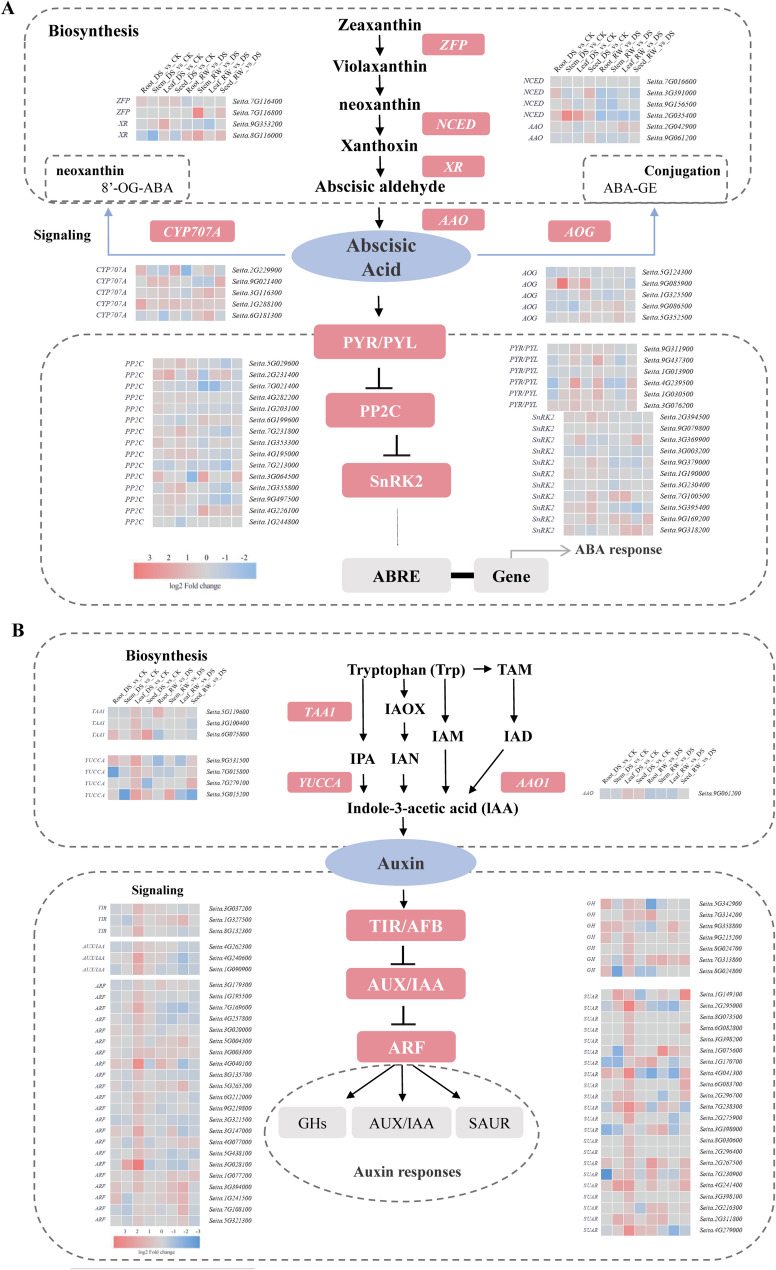
Genes involved in the ABA and IAA biosynthesis and signaling pathways.** A** Differential gene expression involved in ABA biosynthesis and signaling pathways under DS and RW treatment. **B** Differential gene expression involved in IAA biosynthesis and signaling pathways under DS and RW treatment. Up-regulated genes are in red, and down-regulated genes are in blue. The color indicates the value of log2 (FC)

As mentioned above, the drought-responsive and rewatering-responsive DEGs were significantly enriched in hormone pathways, such as ABA and IAA biosynthesis and signaling pathways (Fig. 4). We therefore further analyzed the expression patterns of genes involved in these pathways using our transcriptome data (Fig. 5). The gene (*Seita.2G035400*) encoding 9-cis-epoxycarotenoid dioxygenase (NCED), which catalyzes a key step in ABA biosynthesis, was upregulated in the DS vs. CK comparison, whereas four NCED genes were significantly down-regulated in RW vs. DS of all tissues (Fig. 5A). The gene (*Seita.9G353200*) encoding xanthine dehydrogenase (XR), which participated in ABA synthesis, was up-regulated in DS vs. CK but down-regulated in RW vs. DS in leaves (Fig. 5A). This might help to promote ABA synthesis under DS condition to close stomata and reduce transpiration, and accelerate ABA clearance after RW to avoid excessive stress response. The key ABA receptor genes (*Seita.9G437300*, *Seita.4G239500*, *Seita.1G030500*) encoding pyrabactin resistance 1-like (PYR/PYL) were significantly up-regulated in roots but down-regulated in leaves, and while *protein phosphatase 2C* (*PP2C*) genes (*Seita.5G029600*, *Seita.7G231800*, *Seita.7G213000*, *Seita.2G355800*, *Seita.9G497500*) were also down-regulated in leaves under RW condition (Fig. 5A). This might enhance the ABA sensitivity in the roots while reducing the ABA sensitivity in the leaves, which is conducive to the coordination of the root repair process. The genes (*Seita.2G229900* and *Seita.1G288100*) encoding ABA 8’-hydroxylase (CYP707A), a key enzyme in ABA catabolism, were up-regulated particularly in grains and roots under DS condition (Fig. 5A). This might partially degrade ABA, thereby maintaining the growth and development potential of the roots and grains. However, it was then continuously up-regulated in leaves and grains after RW (Fig. 5A), thereby rapidly reducing whole-plant ABA levels.

The *tryptophan Aminotransferase 1* (*TAA1*) gene (*Seita.6G075800*), which encodes a key enzyme in IAA production, was up-regulated in all tissues under DS condition, but down-regulated under RW condition (Fig. 5B). Genes encoding YUCCA flavin monooxygenases, which are central to auxin biosynthesis, were significantly up-regulated in leaves but generally down-regulated in other tissues under DS condition, while under RW they were significantly down-regulated in leaves and grains but up-regulated in stems conditions (Fig. 5B). This may promote IAA synthesis in leaves during drought stress while reducing it upon rewatering, and concurrently enhance IAA synthesis in stems during rewatering to promote stem elongation and vascular development, thereby supporting water transport and leaf expansion. All the IAA signaling and response genes in leaves, including *transport inhibitor response* (*TIR*), *auxin/indole-3-acetic acid* (*AUX/IAA*), *gretchen hagen 3* (*GH3*), *small auxin-up RNA* (*SUAR*), and *auxin response factors* (*ARF*), were significantly up-regulated under the DS condition but mostly down-regulated under RW condition (Fig. 5B). Three genes (*Seita.5G342900*, *Seita.9G358800*, *Seita.8G024800*) encoding GH3 were up-regulated in roots under DS condition but down-regulated under RW condition (Fig. 5B).

Together, these results reveal distinct and dynamic expression patterns of ABA and IAA pathway genes across tissues and treatments, providing a transcriptional basis for understanding the coordinated regulation of stress defense and recovery in foxtail millet.

### Identification of a multi-tissue responsive hub gene in drought resilience

Based on the observed multi-tissue coordination in drought resistance and post-drought recovery, we hypothesized that conserved hub genes might regulate drought resilience in foxtail millet across tissues. We performed WGCNA to identify such conserved regulators under drought and rewatering. Given the substantial baseline transcriptomic differences among tissues, the global co-expression network showed only moderate correlation (~ 0.5) and was thus unsuitable for detecting core genes.

We therefore adopted a tissue-specific network strategy to reveal core response modules within each tissue. In root, the cyan (112 DEGs), turquoise (712 DEGs), and green (231 DEGs) modules exhibited opposite expression patterns across CK, DS, and RW treatments (Figure S7A, Table S6). In stems, the pink (172 DEGs) and brown (239 DEGs) modules positively correlated with rewatering, whereas the royal blue (68 DEGs), sky blue (47 DEGs), light green (71 DEGs), and salmon (105 DEGs) modules showed a negative correlation with RW in this study (Figure S7B, Table S6). Additionally, the yellow (228 DEGs) module in stem was negatively correlated with DS, while the purple (131 DEGs) module positively correlated with DS. In contrast, the orange (53 DEGs) and dark magenta (25 DEGs) module were positively correlated with CK group (Figure S7B, Table S6). In leaves, the brown (740 DEGs) module presents negative correction with DS, whereas the green (168 DEGs) and turquoise (1686 DEGs) modules show negative and positive with RW, respectively (Figure S7C, Table S6). In grains, the purple (37 DEGs) module shows negative correction with DS but positive correction with RW (Figure S7D, Table S6).

We then intersected the tissue‑specific response genes mentioned above from the four tissues via Venn analysis, and identified two DEGs (*Seita.4G256400* and *Seita.9G137500*) that showed core responsiveness in all tissues (Figure S8A). These two core genes were further intersected with tissue‑conserved gene sets from drought (5 genes) and rewatering (20 genes) conditions (Figure S8B). Only one gene, *Seita.9G137500* (*HPP*), was identified as both differentially expressed under rewatering and conserved across all individual tissues. Alignment against the Uniprot database revealed that *HPP* is evolutionarily conserved across gramineous plants (data not shown). These results indicate that *HPP* might function as a key multi‑tissue responsive hub gene in drought recovery.

### Network analysis of HPP in response to DS and RW

**Fig. 6:**
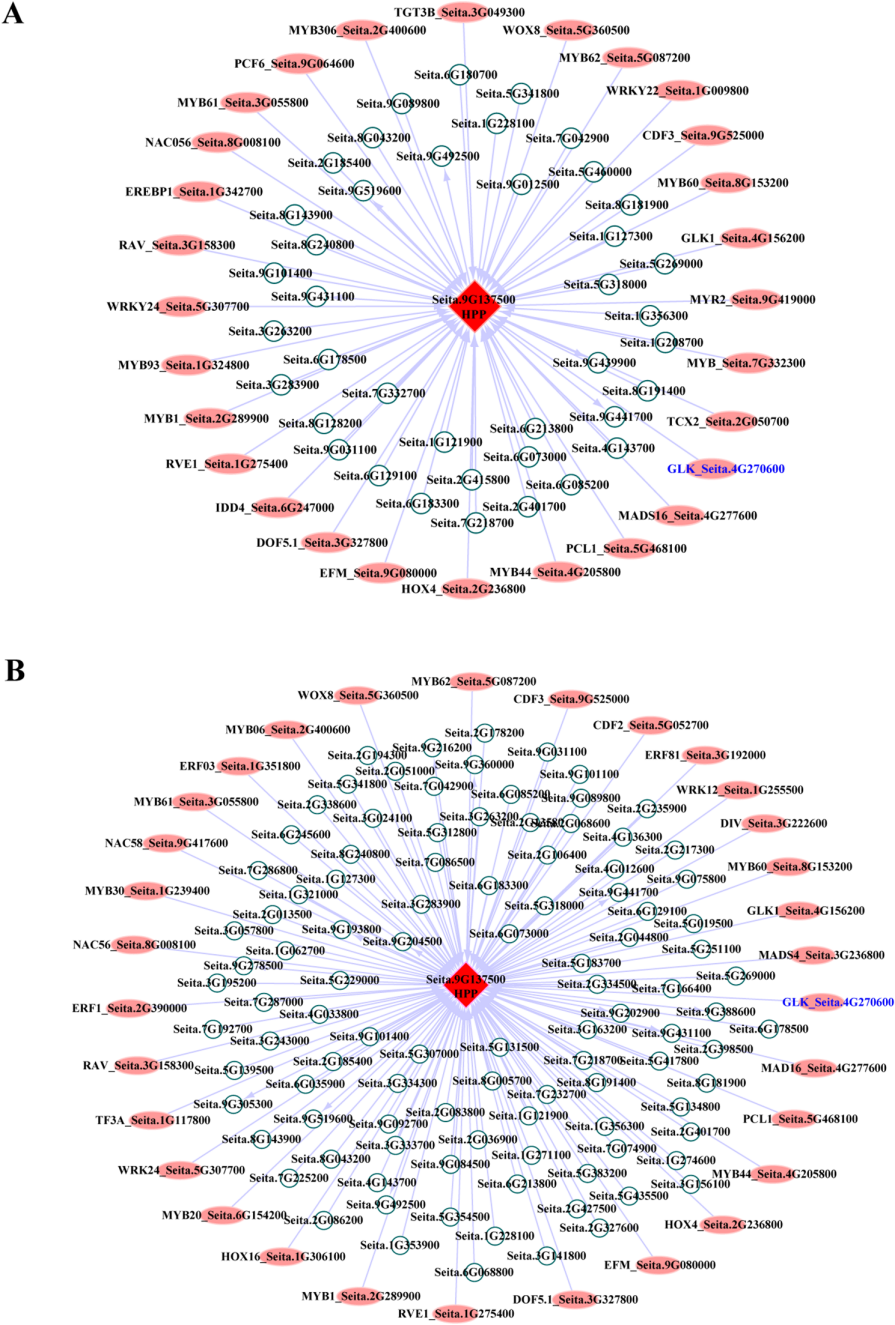
Transcriptional regulatory network analysis for HPP in response to DS and RW treatment. Gene regulatory network maps of HPP with its related DEGs and differentially expressed TFs in response to DS (**A**) and RW (**B**). Diamond indicates HPP, circle indicated related DEGs, and ellipse indicates the predicated TFs for HPP

**Fig. 7:**
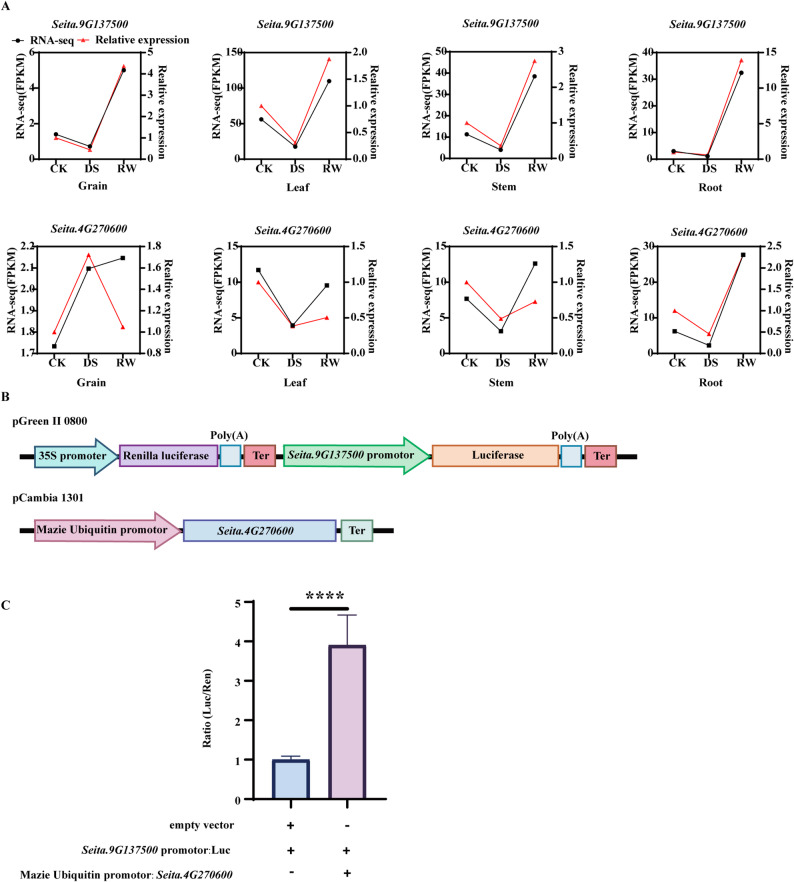
The relationship between HPP and GLK. **A** The gene expression profile of *HPP* and its associated *GLK* in root, stem, leaf, and grain of foxtail millet. The black line represents the RNA-seq results, and the red line represents the qRT-PCR results. **B** Schematic representation of the effectors and reporters used in transcriptional activity analysis of *GLK*-*HPP*. **C** Transactivation assay of *GLK*-*HPP* in protoplast transient assay. Values represent the mean ± SD of six independent experiments. *p*-values were determined using the two-tailed Student’s t-test: ****p < 0.0001

To further investigate the functional role of the *HPP* gene in drought response and post-drought recovery in foxtail millet, we constructed a condition-specific regulatory network centered on *HPP*. Based on gene co-expression analysis, *HPP* expression was found to be correlated with 14, 6, and 28 DEGs in roots, stems, and leaves under DS, respectively. Following RW, *HPP* showed co-expression with 55, 60, 55, and 5 DEGs in roots, stems, leaves, and grains, respectively. In addition, 107 potential TFs were predicted to regulate *HPP*, among which 28 and 31 were differentially expressed under DS and RW, respectively (Table S6). Using these data, we built separate *HPP*-centered regulatory networks for DS and RW conditions (Fig. 6; Table S7-S8). Notably, one predicted TF, *Seita.4G270600* (encoding a Golden2-like/GLK protein, a member of the GARP superfamily of MYB transcription factors), was not only co-expressed with *HPP* under both DS and RW treatments (Fig. 7A), but also bioinformatically predicted to be a potential transcriptional regulator of *HPP*. This dual link suggests that HPP might coordinate drought resistance and rewatering responses across foxtail millet tissues through the upstream regulator *GLK*.

To confirm the reliability of the RNA-seq data, we randomly selected 12 genes (including *HPP* and *GLK*) for qRT-PCR validation across the four tissues. The expression patterns showed strong consistency between qRT-PCR and RNA-seq results (**Figure S9**), supporting the robustness of our sequencing data and subsequent analyses. Intriguingly, *HPP* and *GLK* exhibited nearly identical expression dynamics under both DS and RW conditions. To experimentally test the predicted regulatory relationship, we performed luciferase reporter assays in foxtail millet protoplasts. The results demonstrated that *GLK* strongly binds to the *HPP* promoter, driving luciferase activity approximately fourfold higher than the control (Fig. 7B-C). In summary, our study identifies *HPP* as a conserved, multi‑tissue regulatory factor under drought and rewatering conditions in foxtail millet, and suggests that its role in drought tolerance and recovery is likely mediated through the upstream transcription factor *GLK*. Functional characterization of *HPP* and *GLK* will be addressed in future studies.

## Discussion

Foxtail millet, a traditional drought-tolerant cereal crop in China, plays an irreplaceable role in agricultural production in arid and semi-arid regions [33]. With the increasing severity of global climate change, drought has become one of the major abiotic stresses limiting crop productivity. Therefore, elucidating the drought resistance mechanisms of foxtail millet holds urgent practical significance for breeding water-saving and drought-tolerant crops and ensuring food security in drought-prone areas.

### Multi-tissue coordination: a paradigm shifts from systemic defense to localized repair

In recent years, research on plant drought resistance and rewatering mechanisms has gradually shifted from analyzing individual tissues or gene functions toward dissecting multi-tissue coordinated responses at the systemic level. The functional differentiation and synergy of the “root-stem-leaf” axis observed in foxtail millet in this study align with the “source-sink-flow” redistribution strategy reported in other gramineous crops under drought stress [34–36]. Specifically, the enhanced expression of water- and ion-uptake related genes in roots, coupled with adjusted polar auxin transport to promote deeper root growth under drought (Fig. 4**)**, closely resembles the drought-induced root architecture remodeling mechanisms documented in rice and maize [37–39]. This indicates that optimizing water acquisition under arid conditions is a conserved strategy across species.

Our study further reveals that rewatering is not simply a reversal of drought but an active biological event with distinct temporal characteristics. Sampling at 24 h post-rewatering revealed that even at the maturation stage—when tissues are approaching senescence—the foxtail millet genome remains transiently responsive to water replenishment, providing insight into developmental stage-dependent stress sensitivity. This observation is consistent with the emerging view that drought recovery constitutes a physiological phase independent of the stress response itself [40, 41]. We found that ABA signaling shifts from systemic transport to localized perception and response, while stomatal reopening is delayed after rewatering. It is consistent with the view that the physiological strategy of plants under high temperature stress changes from “growth priority” to “survival priority” based on the integrated analysis of 207 global warming control experiments [42]. Particularly noteworthy is that during rewatering, the photosynthetic apparatus in leaves remains suppressed, whereas repair-related pathways such as photosystem II restoration are preferentially activated. This supports the “repair-before-reactivation” model of photosynthetic recovery [43], and provides transcriptional-level evidence for the hypothesis that chloroplast repair precedes the full recovery of photosynthetic function. Moreover, the sustained activity of ROS scavenging across multiple tissues after rewatering further underscores that oxidative damage repair is a key rate-limiting step in the recovery process [44].

While our transcriptomic data thus provide an extensive reprogramming of hormone and photosynthesis pathways under drought and rewatering conditions, the physiological assays in this study were limited to general stress markers (H_2_O_2_, proline, and MDA) in leaves. This choice was justified, as leaves are the primary site of transpiration and photosynthesis [45, 46]. They also exhibited the most extensive transcriptional changes, making them the most informative tissue for validation (Figs. 3 and 4). Technical constraints also precluded reliable physiological measurements from small organs like grains and roots. Although these data confirmed the establishment of stress, they did not directly quantify specific hormone levels (e.g., ABA) or photosynthetic apparatus. Future studies integrating targeted metabolomics and physiology to directly measure these parameters will therefore be essential to validate the transcriptomic findings.

### Hormonal signaling reprogramming: functional dynamics and synergistic interaction between ABA and IAA

In recent years, the role of plant hormones in drought response and recovery has become a key focus in crop stress resistance research. This study demonstrates that under drought conditions, ABA functions as a core stress signal that activates whole-plant defense responses, consistent with the general pattern of ABA accumulation and signaling upregulation under water stress [47]. However, during early rewatering, the ABA signaling axis undergoes a distinct reprogramming characterized by “reduced systemic transport‑enhanced local perception,” suggesting that ABA may assume roles beyond its conventional stress-signaling function during the water recovery phase. This study further reveals that enhanced local ABA signaling after rewatering coincides temporally with delayed stomatal reopening, activated oxidative stress responses, and photosystem II repair, supporting the hypothesis that ABA participates in coordinating a “repair-first” program during early rewatering. These findings also align with the regulatory mechanisms of ABA metabolism and redistribution during stress relief [48].

Simultaneously, the auxin pathway is widely activated under drought and remains active in its transport and response networks after rewatering, highlighting the central role of this hormone in regulating morphological plasticity. The auxin-mediated remodeling of root architecture and suppression of shoot growth under drought accord with the regulatory function of auxin in root responses to water stress [49]. The rapid adjustment of the auxin network after rewatering may provide immediate signals for morphological reconstruction, such as shifting root growth from deep penetration to lateral root formation and releasing drought-induced inhibitory structures (e.g., leaf rolling) in aerial parts, reflecting the role of auxin in promoting structural rebuilding upon improved water availability [50].

Notably, this study demonstrates that the interaction between ABA and auxin shifts markedly between drought and rewatering phases: transitioning from an antagonistic relationship (growth suppression vs. defense activation) during drought to a synergistic one during rewatering. This dynamic interaction may be linked to cross‑regulation at the transcriptional level between the two hormone signaling pathways, such as the mutual influence of IAA and ABA signaling in regulating stomatal movement and root growth [51]. In tomato, the regulation of ABA on tomato root growth may involve IAA response during soil drying and restoration [52]. These findings further underscore the critical role of “hormone crosstalk” in plant environmental adaptation and offer new perspectives for synchronously improving crop drought tolerance and recovery capacity by modulating key interaction nodes. This study elucidates the regulatory basis underlying multi‑tissue coordinated stress resistance in foxtail millet during drought and rewatering from the perspective of hormone signaling reprogramming and synergistic interaction.

### HPP-GLK module: a potential multi-tissue stress response coordination hub

The HPP family (HPP, Pfam PF04982) proteins, identified in our study, are integral membrane proteins containing a conserved His-Pro-Pro motif and four transmembrane helices. The function of PF04982 – HPP family remains uncertain to our limited knowledge. This family is conserved in cyanobacteria and Arabidopsis, and was firstly identified as playing a key role in nitrite transport [53]. Although direct evidence of its involvement in abiotic stress responses remains insufficient, its functions in nitrogen metabolism, pH adaptation, and tissue-specific expression suggest that it may participate in regulating plant adaptation to environmental pressures such as nitrogen deficiency, soil pH variation, and salt stress. Recent studies have also shown that nitrogen can enhance post-drought recovery in wheat by modulating the *TaSnRK2.10*-*TaNLP7* regulatory module [54], indicating that nitrogen signaling may cooperate with the ABA pathway in regulating post-drought recovery. Given the established role of *HPP* family members in nitrogen metabolism and the recent evidence linking nitrogen signaling to drought recovery, the high conservation of HPP across gramineous plants observed in our study further supports its potential involvement in ABA-regulated post-drought recovery in foxtail millet.

Additionally, *Seita.4G270600* encodes a G2-like transcription factor, which belongs to the GARP superfamily of MYB-type transcription factors. GLK and MYB transcription factors are reported as key regulators of plant drought tolerance, although their roles in post-drought recovery remain poorly documented to our limited knowledge. For example, overexpression of the *ZmGLK* gene in rice enhances drought tolerance by promoting stomatal closure and reducing water loss through leaves via ABA-mediated signaling under drought conditions [55]. *GhMYB102* plays a key role in enhancing drought resistance through its involvement in ABA biosynthesis [56]. Thus, we speculate that the *GLK*-*HPP* module may jointly regulate drought tolerance and post-drought recovery in foxtail millet through ABA-mediated signaling. This hypothesis warrants further experimental validation. If confirmed, this module could not only provide a novel molecular target for stress-resistance breeding in foxtail millet but also enhance our understanding of the systemic stress-regulatory networks in gramineous crops.

## Conclusions

In this study, we systematically uncover the dynamic, multi-tissue regulatory framework underlying drought adaptation and post‑rewatering recovery in foxtail millet. Physiological and transcriptomic analyses revealed that prolonged drought induces oxidative stress and osmotic adjustment, while short-term rewatering rapidly restores homeostasis. Global gene expression reprogramming highlighted the central roles of hormone signaling and photosynthetic regulation throughout these transitions. Importantly, we identified a conserved, drought- and rewatering-responsive gene module, *HPP*-*GLK*, which appears to function as an integrative hub coordinating stress perception and tissue-specific responses across root, stem, leaf, and grain tissues. These findings not only advance our understanding of the molecular basis of drought resilience in millet but also provide a promising target module for simultaneously enhancing drought tolerance and post-stress recovery in cereal crops through molecular breeding.

## Supplementary Information

Supplementary Material 1.

Supplementary Material 2.

## Data Availability

The raw sequence reads are available for download from the China National Center for Bioinformation (CNCB) Genome Sequence Archive (GSA) database (CRA037659).

## Abbreviations

CK: Control group
DS: Drought stress group
RW: Rewater group
DEGs: Differentially expressed genes
GO: Gene ontology
TF: Transcription factor
GLK: Golden2-like
