# Supplementary material for: Multi-tissue transcriptomic profiling of foxtail millet in response to drought and rewatering
Source: BMC Plant Biol. 2026 May 11;26:1124. doi: 10.1186/s12870-026-08906-y (PMC13330349; doi:10.1186/s12870-026-08906-y)
Supplement: Supplementary file 2 — Supplementary Material 2. [file 12870_2026_8906_MOESM2_ESM.docx]

**Supplementary Figures**

**Figure S1. Relative humidity and root morphology during drought stress and rewatering treatment in foxtail millet.**

**Figure S2**. **Gene expression in different tissues of foxtail millet in response to drought stress and rewatering.**

**Figure S3. Density distribution of gene expression levels.**

**Figure S4.** **Gene ontology (GO) enrichment map of DEGs at all the tissues of different treatments.**

**Figure S5. Biotic stress pathways showing differential expression in different tissues under drought stress or rewatering.**

**Figure S6. Metabolic pathways showing differential expression in different tissues under drought stress or rewatering.**

**Figure S7. The heatmap of the correlation between module and treatments from WGCNA.**

**Figure S8. Venn diagrams of the DEGs among different groups.**

**Figure S9. Validation of the expression levels of selected genes obtained from RNA-seq using qRT-PCR.**

**Figure S1. Relative humidity and root morphology during drought stress and rewatering treatment in foxtail millet. (A)** Dynamic changes in relative humidity in the growing environments for control (CK), drought-stressed (DS), and rewatered (RW) plants throughout the experimental period. Day 0 (D0, August 27, 2025), most foxtail millet plants entered the booting stage. D20 (September 15, 2025), all foxtail millet plants had fully booted. D30 (September 26, 2025), the relative soil humidity content in the DS and RW groups decreased to 20~30%. D50 (October 15, 2025), the RW group was subjected to rewatering treatment for one day. **(B)** Root morphology of foxtail millet under drought stress and rewatering treatment. Bar = 10 cm.

**Figure S2**. **Gene expression in different tissues of foxtail millet in response to drought stress and rewatering.** Bar graphs of total number of genes expressed (based on TPM) in different tissues/treatments of foxtail millet.

**Figure S3. Density distribution of gene expression levels.** Expression levels for all genes across individual samples from root, stem, leaf, grain under CK, DS and RW treatments.

**Figure S4.** **Gene ontology (GO) enrichment map of DEGs at all the tissues of different treatments.** Significantly enriched GO terms in DS and/or RW were overlapped to highlight the terms specifically or commonly in DS and/or RW. Blue border in the circle, GO terms specific to DS. Yellow border in the circle, GO terms specific to RW. Red border in the circle, GO terms common. The larger the circle, the greater the number of genes annotated to this GO pathway.

**Figure S5. Biotic stress pathways showing differential expression in different tissues under drought stress or rewatering.** DEGs (fold change ≥2, q-value ≤ 0.05) in DS vs. CK and RW vs. DS of root, stem, leaf, and grain were loaded into MapMan to generate the overview. On the log2 scale, red colors represent upregulation (DS vs CK; RW vs DS), and blue indicates downregulation (DS vs. CK; RW vs. DS).

**Figure S6. Metabolic pathways showing differential expression in different tissues under drought stress or rewatering.** DEGs (fold change ≥2, q-value ≤0.05) in DS vs. CK and RW vs. DS of root, stem, leaf, and grain were loaded into MapMan to generate the overview. On the log2 scale, red colors represent upregulation (DS vs CK; RW vs DS), and green indicates downregulation (DS vs. CK; RW vs. DS).

**Figure S7. The heatmap of the correlation between module and treatments from WGCNA.** The correlation between module and treatments in root (**A**), stem (**B**), leaf (**C**), and grain (**D**). Red and blue represent positive or negative correlations of modules and traits, respectively. The left column lists the number of genes in each module. The upper number in each square represents the correlation coefficient about modules with different groups, and the lower number in the bracket means *P*-value.

**Figure S8. Venn diagrams of the DEGs among different groups. A** Venn diagrams of the DEGs sets in root, stem, leaf, and grain from WGCNA. **B** Venn diagrams of the common DEGs from Figure 3B, 3C and S9A.

**Figure S9. Validation of the expression levels of selected genes obtained from RNA-seq using qRT-PCR.**
